# Comparative effects of a glucose–fructose bar, glucose–fructose hydrogel and maltodextrin gel on carbohydrate oxidation and sprint performance in Tier 2 athletes

**DOI:** 10.1113/EP093136

**Published:** 2025-11-26

**Authors:** Ewan Dean, Ash Osborne, Daren Subar, Paul Hendrickse, Christopher J. Gaffney

**Affiliations:** ^1^ Lancaster University Medical School Lancaster University Lancaster Lancashire UK; ^2^ BRIDGES Research Group, Department of Surgery East Lancashire Hospitals NHS Trust Blackburn Lancashire UK

**Keywords:** carbohydrate metabolism, exercise, nutrition, oxidation, supplements

## Abstract

Carbohydrate supplementation optimises athletic performance, but the metabolic and performance impacts of commercial products/compositions are underexplored. We compared the efficacy of three commercial carbohydrate supplements: a glucose–fructose bar (GF‐Bar), a glucose–fructose hydrogel (GF‐Gel) and a maltodextrin‐based gel (MD‐Gel). Antegrade venous blood samples for glucose and insulin were measured alongside substrate utilisation in healthy Tier 2 athletes after ingesting 45 g of carbohydrates from the GF‐Bar, GF‐Gel and MD‐Gel during a modified 1‐h oral glucose tolerance test (OGTT). Additionally, the effect of supplementation on high‐intensity interval exercise was evaluated during repeated maximal sprint performance. During the OGTT, the GF‐Bar elicited greater total carbohydrate oxidation than MD‐Gel (24.6 ± 7.4 g vs. 17.8 ± 8.6 g, *P* = 0.038) but not GF‐Gel (20.1 ± 6.4 g, *P* > 0.05). Carbohydrate oxidation per minute varied over time (*P* < 0.001) and between products (*P* = 0.043), with GF‐Bar (0.27 ± 0.05 g min^−1^) showing higher oxidation than GF‐Gel (0.21 ± 0.05 g min^−1^) and MD‐Gel (0.19 ± 0.06 g min^−1^). No differences were observed in glucose peak, time to peak glucose or insulin concentration (*P* > 0.05). Peak power (*P* = 0.011), mean power (*P* < 0.001) and total work varied across sprints (*P* < 0.001) but not between products (*P* > 0.05). Perceived exertion and gastrointestinal discomfort were similar between products (*P* > 0.05). Despite differences in carbohydrate oxidation during the OGTT, the GF‐Bar, GF‐Gel and MD‐Gel displayed similar metabolic and sprint performance outcomes, suggesting that, within this study, carbohydrate formulation did not impact short‐duration maximal exercise.

## Abstract (highlights)

- **What is the central question of this study?**
  Carbohydrate supplementation optimises athletic performance, but the metabolic and performance impacts of commercial products/compositions are underexplored: what is the efficacy of a glucose–fructose bar, a glucose–fructose hydrogel and a maltodextrin‐based gel?
- **What is the main finding and its importance?**
  In Tier 2 athletes, a glucose–fructose bar led to significantly greater total carbohydrate oxidation than a maltodextrin gel during a 1‐h OGTT but not compared to a glucose–fructose hydrogel. No significant effects were observed on blood glucose, insulin, sprint performance or gastrointestinal comfort. All supplements produced similar outcomes during repeated high‐intensity sprints, suggesting that carbohydrate formulation did not influence short‐duration maximal exercise performance in this context.

## 1 INTRODUCTION

Carbohydrate availability is a key determinant of exercise performance, as both circulating glucose and muscle glycogen provide fuel for moderate to high‐intensity activity (Burke et al., 2013; Cermak & van Loon, 2013). Carbohydrate availability refers to the body's endogenous stores of glycogen and blood glucose, which are finite and may limit performance during prolonged or repeated efforts (Burke et al., 2013). Carbohydrate supplementation refers to the exogenous provision of carbohydrate (Stellingwerff & Cox, 2014), often achieved in the form of convenient supplements, typically through gels, drinks and bars composed of easily digestible monosaccharides (e.g., glucose and fructose), disaccharides (e.g., sucrose) and specific high glycaemic index polysaccharides (e.g., maltodextrin) (Baker et al., 2015; Jeukendrup, 2014, 2010).

It is well‐documented that consuming carbohydrates before exercise increases carbohydrate oxidation and enhances the availability of readily oxidisable fuel. Higher carbohydrate oxidation is beneficial as it supports ATP production, helping to fuel muscle contractions and delay fatigue (Hargreaves & Spriet, 2020). During prolonged moderate to high‐intensity steady‐state exercise such as marathon running or long‐distance cycling, carbohydrate supplementation provides an exogenous source of carbohydrate, helping to prevent hypoglycaemia and glycogen depletion (Podlogar & Wallis, 2022). While much of the literature has focused on the ergogenic effects of carbohydrate ingestion during prolonged exercise, evidence suggests carbohydrate intake before high‐intensity intermittent exercise, for example, repeated sprints in football or high‐intensity interval training (HIIT), may offer performance benefits through distinct mechanisms (Paris et al., 2021; Rollo et al., 2020; Vigh‐Larsen et al., 2021; Vigh‐Larsen, Ørtenblad, Emil Andersen, et al., 2022; Vigh‐Larsen, Ørtenblad, Nielsen, et al., 2022).

Repeated high‐intensity exercise relies on phosphocreatine resynthesis and anaerobic glycolysis (Vigh‐Larsen et al., 2021; Vigh‐Larsen, Ørtenblad, Emil Andersen, et al., 2022; Vigh‐Larsen, Ørtenblad, Nielsen, et al., 2022). These processes are sensitive to carbohydrate availability, suggesting a potential role for supplementation in this context. It is suggested that carbohydrate ingestion before repeated high‐intensity exercise performance may have a greater influence on central and peripheral factors, due to the primary fuel source being phosphocreatine and intramuscular glycogen, rather than circulating glucose (Vigh‐Larsen et al., 2021; Vigh‐Larsen, Ørtenblad, Emil Andersen, et al., 2022; Vigh‐Larsen, Ørtenblad, Nielsen, et al., 2022). Centrally, elevated blood glucose helps preserve cognitive function and enhance central drive or motivation during repeated high‐intensity exercise (Nybo, 2003; Paris et al., 2021; Rollo et al., 2020). Peripherally, elevated circulating glucose and insulin support anaerobic glycolytic flux, optimising muscle glycogen availability, contributing to greater total work and attenuated fatigue during maximal efforts (Paris et al., 2021; Rollo et al., 2020). Carbohydrate ingestion during repeated high‐intensity exercise maintained blood glucose (5.3 ± 0.2 vs. 4.1 ± 0.2 mmol L^−1^) and prevented the post‐exercise decline in plasma insulin observed with placebo, resulting in approximately fivefold higher insulin concentrations, suggesting enhanced carbohydrate oxidation and liver glycogen preservation to delay fatigue. However, this did not translate into improved exercise performance (Vigh‐Larsen et al., 2024). Carbohydrate ingestion improved repeated maximal sprint cycling performance, showing higher mean power output (659.3 ± 103.0 vs. 645.8 ± 99.7 W), total work (9849.8 ± 1598.8 vs. 9447.5 ± 1684.9 J) and a lower fatigue index (15.3 ± 8.6 vs. 17.7 ± 10.4 W s^−1^) (Krings et al., 2017).

Different carbohydrate supplements vary in form and composition, such as glucose–fructose mixtures, known to enhance absorption via distinct intestinal transporters: sodium–glucose cotransporter 1 (SGLT1) for glucose and glucose transporter 5 (GLUT5) for fructose (Jentjens et al., 2004; Pfeiffer et al., 2010). This strategy, including combinations like maltodextrin and fructose, has been shown to significantly increase carbohydrate oxidation rates up to 120 g h^−1^ during prolonged endurance exercise (Hearris et al., 2022; Pfeiffer et al., 2010; Podlogar & Wallis, 2022; Podlogar et al., 2022; Wallis et al., 2005). Despite these findings, limited research has compared different carbohydrate formulations consumed before short‐duration, maximal exercise. Most available supplements – gels, bars and hydrogels – contain distinct carbohydrate compositions (maltodextrin/glucose–fructose blends) which may affect absorption, glycaemic response and subsequent energy availability (Guillochon & Rowlands, 2017; Jeukendrup, 2014; King et al., 2020; Podlogar & Wallis, 2022; Rowe et al., 2022; Saunders et al., 2007). This variety can overwhelm consumers, particularly as carbohydrate intake should be tailored to exercise duration, intensity and individual preference (Jeukendrup, 2014; Podlogar & Wallis, 2022). Furthermore, whether these compositional differences translate to meaningful physiological or performance outcomes in the context of short, high‐intensity exercise remains unclear.

Evidence for an acute ergogenic effect of carbohydrate supplementation on short‐duration, high‐intensity efforts is inconsistent, with some research supporting improved performance (Cooper et al., 2014; Krings et al., 2017) and others reporting no changes in performance (Gough et al., 2022; McMahon & Thornbury, 2020; Vigh‐Larsen et al., 2024), and less is known about whether different carbohydrate formulations elicit distinct metabolic or performance responses. This represents an important gap in the literature given the widespread pre‐competition use of commercial carbohydrate supplements by athletes, even in short‐duration, high‐intensity activities. The present study aimed to compare the postprandial glucose and metabolic responses of three commercially available carbohydrate supplements at rest and during high‐intensity exercise, and to explore their potential influence on repeated sprint performance, in order to determine whether formulation‐specific differences exist despite the limited ergogenic effects typically reported in such exercise contexts. Two complementary trials were conducted to provide both mechanistic and applied insight into the effects of differing commercially available carbohydrate supplements. An oral glucose tolerance test (OGTT) was performed under resting conditions to tightly control external variables and determine differences in substrate metabolism between products. The subsequent exercise trial was designed to examine whether the metabolic differences observed at rest translated into alterations in repeated high‐intensity exercise.

This research can help athletes make informed decisions about the effectiveness of their nutritional supplements. The carbohydrate supplements compared in this study were the glucose–fructose Voom Pocket Rocket Electro Energy bar (VOOM Nutrition, Yealand Redmayne, UK) (GF‐Bar), the glucose–fructose hydrogel Maurten Gel 160 (Maurten, Gothenburg, Sweden) (GF‐Gel) and the maltodextrin‐based Science in Sport Go Isotonic Energy gel (Science in Sport, London, UK) (MD‐Gel).

## 2 METHODS

### 2.1 Ethical approval

In both studies, subjects were fully informed of all procedures and potential risks before providing written informed consent. All protocols were approved by the Lancaster University Ethics Committee: OGTT ethics number: LMS‐24‐Dean‐1; and Exercise trial ethics number: LMS‐24‐Dean‐2. Both studies were conducted in accordance with the *Declaration of Helsinki*, 8th Revision, and Good Clinical Practice. Both studies used a randomised crossover design and were preregistered on clinicaltrials.gov: OGTT trial (NCT06375577) and Exercise trial (NCT06768333).

### 2.2 Subjects

All subjects were classified as Tier 2 athletes (McKay et al., 2021), indicating they trained regularly (∼3 times per week) to compete in their respective sports, including running, cycling or triathlon. Subjects were recruited through local sports clubs, social media and posters. They were contacted to discuss the study and eligibility criteria, and, if deemed suitable, provided with an information sheet and an in‐person screening date. Subjects’ anthropometric characteristics are detailed in Table 1. The modified oral glucose tolerance test (OGTT) was designed as a lab‐based assessment of metabolic responses. Given evidence that substrate metabolism varies by sex due to hormonal differences between males and females (Cano et al., 2022; Ciarambino et al., 2023; Tucker et al., 2025), and that females may exhibit higher rates of non‐oxidative free fatty acid clearance at rest, which can influence the respiratory quotient (RQ) (Sanchez et al., 2024), only males were recruited. This methodological decision was taken to reduce heterogeneity and improve internal validity, consistent with recommendations for mechanistic studies where experimental control is prioritised (Berg et al., 2025).

In contrast, the exercise trial prioritised ecological validity by including both male and female athletes to better reflect the applied nature of the study design and real‐world sporting contexts. Notably, all five females recruited for the exercise study reported hormonal contraceptive use, which can stabilise menstrual cycle fluctuations and yield more consistent metabolic and performance responses compared to naturally menstruating females (Elliott‐Sale et al., 2020; Smith et al., 2024). To ensure consistency across the two trials, the five males in the exercise study also participated in the OGTT.

**TABLE 1:** Anthropometric characteristics.

Subjects	OGTT trial (16 males)	Exercise trial (5 males)	Exercise trial (5 females)	Exercise trial (total)
Age (years)	23 ± 4.2	27.8 ± 5.7	23.2 ± 1.8	25.5 ± 4.7
Height (cm)	182.0 ± 6.5	181.9 ± 3.4	170.7 ± 6.2	176.3 ± 7.6
Weight (kg)	79.5 ± 8.3	80.4 ± 7.8	68.1 ± 8.9	74.3 ± 10.3
BMI (kg m^−2^)	23.81 ± 1.2	23.8 ± 1.3	22.7 ± 2.1	23.2 ± 10.3
Lean mass (kg)	65.8 ± 5.4	65.6 ± 3.9	48.3 ± 3.1	56.9 ± 9.7
Body fat (%)	14.5 ± 5.0	13.7 ± 3.5	24.6 ± 5.7	19.2 ± 7.3

### 2.3 Sample size justification

For the OGTT, an a priori power analysis (G*Power 3.1) for a repeated measure, within‐subjects ANOVA (1 group, 3 levels), *f* = 0.40, 80% power, *r* = 0.50 (ε = 1), α = 0.016 (Bonferroni correction for three supplements), 16 subjects were required. The OGTT is typically employed in clinical populations to assess glucose regulation (Jagannathan et al., 2020), with little work examining its application in healthy athletic cohorts. The OGTT was conducted at rest with no prior exercise, so *f* = 0.40 was selected as a conservative estimate of a moderate, physiologically meaningful difference in post‐prandial glucose in healthy Tier 2 athletes, in whom within‐subject variability is expected to be low. While one study (Flockhart et al., 2023) demonstrates exercise can modulate glucose tolerance during an OGTT, our resting OGTT provides a clearer, conservative assessment of carbohydrate supplements without confounding by prior exercise.

For the exercise trial, an a priori power analysis (G*Power 3.1) for a repeated measure, within‐subjects ANOVA (1 group, 3 levels), *f* = 0.30, 80% power, *r* = 0.50 (ε = 1), α = 0.016, required 10 subjects. This effect size was chosen for glucose, reflecting mixed findings in prior research on repeat maximal exercise (Dorling & Earnest, 2013; Krings et al., 2017; Vigh‐Larsen et al., 2024), where carbohydrate ingestion produced trivial to moderate effects on metabolism and performance. Accordingly, *f* = 0.30 was adopted as a conservative, physiologically realistic estimate, with performance treated as a secondary outcome.

### 2.4 Medical screening

All subjects completed a medical screening form aligned with the American College of Sports Medicine (ACSM) safety‐to‐exercise guidelines (Liguori & American College of Sports Medicine, 2020) to confirm no contraindications to exercise or allergies to the carbohydrate products, as previously described in our lab (Gaffney et al., 2022). To be included in the study, subjects were required to have a body mass index (BMI) of 18.5–24.9 kg m^−2^ based on NHS‐defined healthy ranges and prior research on athletes’ BMI (Heller et al., 2022; Knechtle et al., 2011; Marc et al., 2014). Subjects were excluded if they had any diagnosed medical condition, took prescribed medication, or adhered to diets (such as high‐carbohydrate–low‐fat or low‐carbohydrate high‐fat) that affected gut microbiome glucose responses (Rauch et al., 2022). Height and body mass were measured using an ultrasonic stadiometer and scales (217 ultrasonic stadiometer and scales, Seca, Hamburg, Germany), while body composition was assessed with bioimpedance scales (DC‐430P, Tanita, Tokyo, Japan). These measures were collected, as inter‐individual variation in anthropometry can influence substrate metabolism and exercise performance (Ijaz et al., 2024). To ensure subjects were safe to exercise, blood pressure was measured using an automatic blood pressure monitor (M3 Comfort, Omron, Kyoto, Japan).

### 2.5 Experimental protocols

Both the OGTT and exercise study were conducted following a researcher‐blinded randomised crossover design and required three experimental visits each, separated by a minimum of 48 h to replicate the frequency of Tier 2 athletes’ training. Subjects were asked to continue their typical diet in the days leading up to each visit. Subjects were required to refrain from caffeine 24 h before each visit and were only permitted to drink water on the day of each visit. For the exercise study, female participants reported their contraceptive use. All five female participants were using hormonal contraceptives.

### 2.6 OGTT trial

For the OGTT trial, 16 healthy male Tier 2 athletes attended the Human Performance Laboratory at Lancaster University following a 2‐h fast and were asked to replicate their breakfast meal for subsequent visits. Upon arrival, an antegrade venous cannula (Vasofix Safety IV Catheter 18G, BBraun, Sheffield, UK) was inserted into the antecubital fossa of the forearm, and a resting blood sample of 1 mL was drawn for blood glucose, lactate and electrolytes (sodium, potassium, chloride), and a 3 mL sample was taken for insulin using a gold‐top serum separator vacutainer (CAT Serum Sep Clot Activator, VACUETTE, Greiner Bio‐One, Stonehouse, UK). Subjects were then seated semi‐supine on a medical bed (Plinth 2000 Medical Bed, Plinth Medical, Occold, UK) and rested quietly for 15 min to allow them to reach a relaxed state, as previously described in research measuring resting metabolic rate (Blannin & Wallis, 2024). To prevent clotting, the cannula was flushed with ∼1 mL 0.9% saline every 15 min. A flush log was maintained, and on average, 4.6 ± 0.7 mL of saline was used per visit. A small sample was discarded after each flush to ensure the cannula was fully primed with blood before a sample was taken.

After the 15‐min rest period, subjects consumed 45 g of carbohydrates from either the GF‐Bar, or GF‐Gel, or MD‐Gel. A 60‐min OGTT was used to reflect the comparatively lower carbohydrate dose (45 g vs. 75 g for standard OGTT) (Jagannathan et al., 2020), typical of pre‐exercise consumption, and to align with real‐world timeframes in which athletes ingest carbohydrates before exercise (Thomas et al., 2016). During the 1‐h OGTT, blood was sampled via an antegrade venous cannula at regular intervals. One millilitre was collected every 5 min for blood glucose, lactate and electrolytes. Three millilitres were collected every 10 min for insulin. Substrate utilisation was measured via indirect calorimetry using breath‐by‐breath analysis, recording the RQ via a face mask (Hans Rudolph 7450, Hans Rudolph, Shawnee, KS, USA) connected to an online gas analyser (Cortex Metalyzer 3B‐R3, Cortex, Leipzig, Germany).

Carbohydrate and fat oxidation were calculated using the Frayn (1983) equations, which are appropriate for resting conditions where steady state assumptions apply:

$$\begin{array}{ccc} \mathrm{Carbohydrate}\;\mathrm{oxidation}\left(g\mathrm{mi}n^{-1}\right) & = & 4.55\times {\dot{V}}_{CO_{2}}\left(L\mathrm{mi}n^{-1}\right)\\ & & -\;3.21\times {\dot{V}}_{O_{2}}\left(L\mathrm{mi}n^{-1}\right) \end{array}$$

$$\mathrm{Fat}\mathrm{oxidation}\left(g\mathrm{mi}n^{-1}\right)=1.67\times {\dot{V}}_{O_{2}}\left(L\mathrm{mi}n^{-1}\right)-1.67\times {\dot{V}}_{CO_{2}}\left(L\mathrm{mi}n^{-1}\right)$$

Carbohydrate oxidation efficiency was estimated as the percentage of the ingested carbohydrate (45 g) that was oxidised, by dividing the total carbohydrate oxidised by 45 and multiplying it by 100. This approach provides a good estimation in the absence of ^13^C tracer data (Hulston et al., 2009).

### 2.7 Exercise visits

For the exercise trial, 10 healthy Tier 2 athletes (*n* = 5 females, *n* = 5 males) consumed a high‐carbohydrate control meal of 196 kcal, consisting of 64% carbohydrate (27.0 g, of which sugars 11.3 g), 18% fat (7.6 g) and 8% protein (3.6 g), with 2.7 g fibre and 0.36 g salt 2 h before attending the Human Performance Lab to mimic pre‐training or competition nutrition (Thomas et al., 2016). As with the OGTT, an antegrade venous cannula was inserted into the antecubital fossa of the forearm. Subjects consumed 45 g of carbohydrates from the GF‐Bar, GF‐Gel or MD‐Gel 35 min before the start of exercise, as the mean time of peak glucose availability identified in the OGTT trial.

### 2.8 Repeated sprint protocol

Following a 3‐min warm‐up cycling at 70 rpm, subjects completed five 15‐s maximal sprints against 0.075 kg kg^−1^ body mass, interspersed with 3 min of active recovery at 70 rpm against no resistance. In accordance with the ACSM guidelines (Liguori & American College of Sports Medicine, 2020), the exercise was followed by a supervised active cool‐down period, during which subjects continued low‐intensity cycling. Heart rate was continuously monitored using a chest‐worn heart rate monitor (Polar H10, Polar, Kempele, Finland), and subjects remained under observation until their heart rate returned to within 20% of pre‐exercise resting values. Gastrointestinal (GI) discomfort was recorded before and after exercise via a modified version of the Gastrointestinal Symptom Rating Scale (GSRS), shown to have good test–retest reliability in athletes (Wardenaar et al., 2024). Subjects reported their ratings of perceived exertion (RPE) after the warm‐up and after the final sprint using Borg's 6–20 RPE scale (Borg, 1982). One millilitre of blood was sampled via an antegrade venous cannula for blood glucose, lactate and electrolytes (sodium, potassium, chloride and calcium) at baseline, after the warm‐up, and then at the end of each sprint and 3‐min recovery period. Substrate utilisation was measured via indirect calorimetry using breath‐by‐breath analysis, recording the respiratory exchange ratio (RER) via a face mask (Hans Rudolph 7450) connected to an online gas analyser (Cortex Metalyzer 3B‐R3), and heart rate was recorded throughout.

### 2.9 Subject randomisation and blinding procedures

**FIGURE 1:**
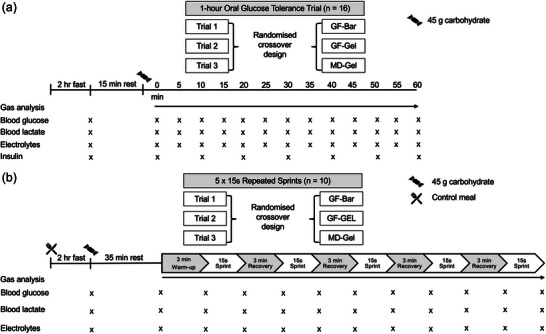
Flowchart study design and schematic of the 1‐h oral glucose tolerance trial (OGTT) (a) and repeated intermittent sprint intervals (b).

Supplements were randomised by the laboratory technician using an online randomisation tool (Research Randomiser: https://www.randomizer.org). Carbohydrate products were labelled A, B and C, with product details sealed in an envelope stored in a locked cabinet. This envelope would only be opened if an adverse reaction occurred. No adverse events were reported, so blinding remained intact until analysis was complete. The study utilised a randomised, researcher‐blinded crossover design. To reduce bias, subjects were not informed of product names, and the researcher responsible for data collection and analysis remained blinded to allocation until all analyses were complete. Although the physical form of the products (bar, gel, hydrogel) became apparent upon consumption, randomisation and blinding procedures were implemented to minimise researcher bias. Figure 1a shows the study design for the OGTT, and Figure 1b for the exercise trial.

### 2.10 Supplement administration

All supplements were prepared by a laboratory technician in accordance with UK food hygiene standards. Supplements were provided in a clear plastic bowl. For both trials, the laboratory technician weighed the supplements before and after consumption to ensure all 45 g of carbohydrates had been consumed. This equates to 47 g of the GF‐Bar, 73.13 g of the GF‐Gel and 122.7 g of the MD‐Gel. For the OGTT, subjects consumed 49.94 ± 2.79 g of the GF‐Bar, 74.56 ± 1.55 g of the GF‐Gel and 122.81 ± 1.28 g of the MD‐Gel. For the exercise study, subjects consumed 48.4 ± 1.78 g of GF‐Bar, 75.22 ± 1.55 g of GF‐Gel and 122.3 ± 1.49 g of MD‐Gel. Nutritional information is shown in Table 2.

**TABLE 2:** Nutritional information of study supplements.

Supplement	Ingredients	Nutritional information (matched for 45 g carbohydrate)
GF‐Bar	Raw cane sugar, glucose syrup, water, dried fruit (1%), electrolytes (tri‐sodium citrate, pink Himalayan salt, potassium chloride, magnesium oxide, calcium lactate) (0.3%), natural flavouring, B‐vitamins. No artificial sweeteners, thickeners or preservatives	176 kcal, 45 g carbohydrates, 41 g of which sugar, 0 g fat, 0 g protein, trace salt, 1 mg B‐vitamins, 120 mg electrolytes
GF‐Gel	Water, glucose, fructose, gelling agent: calcium carbonate, gelling agent: gluconic acid, gelling agent: sodium alginate	180 kcal, 45 g carbohydrates, 45 g of which sugars, 0 g fat, 0 g protein, 90 mg salt
MD‐Gel	Water, maltodextrin (from maize) (33%), gelling agents (gellan gum, xanthan gum), natural flavouring, acidity regulators (citric acid, sodium citrate), preservatives (sodium benzoate, potassium sorbate), sweetener (acesulfame K), sodium chloride, antioxidant (ascorbic acid)	178 kcal, 45 g carbohydrates, 1.23 g of which sugars, 0 g fat, 0 g protein, 20 mg salt

### 2.11 Outcomes

#### 2.11.1 Blood analysis

Blood glucose and lactate were analysed immediately using a bench‐top blood analyser (Biosen C‐Line GP+, EKF, Barleben, Germany), and electrolytes were analysed using a bench‐top electrolyte analyser (i‐smart 30 PRO, Woodley Laboratory Diagnostics, Bolton, UK).

#### 2.11.2 Insulin analysis

For insulin, the 3 mL vacutainer was inverted several times and left to clot at room temperature for 15 min before being centrifuged at 4°C, 1800 RCF, for 10 min. The supernatant was then transferred to a microfuge tube and stored at −20°C during the study visit before moving to −80°C for analysis at a future date. Insulin was measured using an enzyme‐linked immunosorbent assay (ELISA) (Human Insulin ELISA Kit, CrystalChem, Elk Grove Village, IL, USA). Samples were prepared and analysed, and absorbance was read at both 450 nm and 630 nm before subtracting the 630 nm absorbance readings from the 450 nm absorbance readings, following the manufacturer's protocol. Insulin concentrations are presented as micro‐units per millilitre. Missing insulin values (GF‐Bar = 7.8%, GF‐Gel = 8.6%, MD‐Gel = 6.3%) were imputed using a Monte Carlo method to minimise bias from incomplete observations (Austin & van Buuren, 2022; Dong & Peng, 2013; Schafer, 1997).

To enable temporal comparisons between glucose and insulin, values were normalised to a 0%–100% scale based on the range of each data set (where 0% represented the lowest value and 100% the highest value), as previously described (Atherton et al., 2010). Normalisation reflected each subject's percentage change from baseline at 10‐min intervals during the OGTT, using the following equation:

$$\begin{array}{ccc} & & \left(\left[\mathrm{Current}\;\mathrm{Value}-\mathrm{Baseline}\;\mathrm{Value}\right]/\right.\\ & & \;\left.\left[\mathrm{Maximum}\;\mathrm{Value}-\mathrm{Minimum}\;\mathrm{Value}\right]\right)\times 100 \end{array}$$

Following normalisation, the relative changes in glucose and insulin over time were compared to examine the temporal dynamics of both variables across the 1‐h OGTT.

#### 2.11.3 Statistical analysis

Data normality was assessed using the Shapiro–Wilk test. Results are reported as means ± SD unless stated otherwise. Normally distributed time‐series data were analysed using two‐way repeated measures ANOVA with product and time as within‐subject factors. Significant main or interaction effects were followed up with a Tukey multiple comparison test. Non‐parametric data were analysed using Friedman's test, with Dunn's *post hoc* test for significant effects. The area under the curve was calculated for glucose and insulin responses using the trapezoidal method in GraphPad Prism 10.4.1 (GraphPad Software, Boston, MA, USA). Raw concentrations were plotted against time, and the total area under the curve was determined for each participant across the 60‐min OGTT. Data analysis and figure preparation were conducted using GraphPad Prism 10.4.1. Statistical significance was set at *P* < 0.05.

## 3 RESULTS

### 3.1 Resting OGTT

#### 3.1.1 Blood glucose and insulin responses to OGTT

**FIGURE 2:**
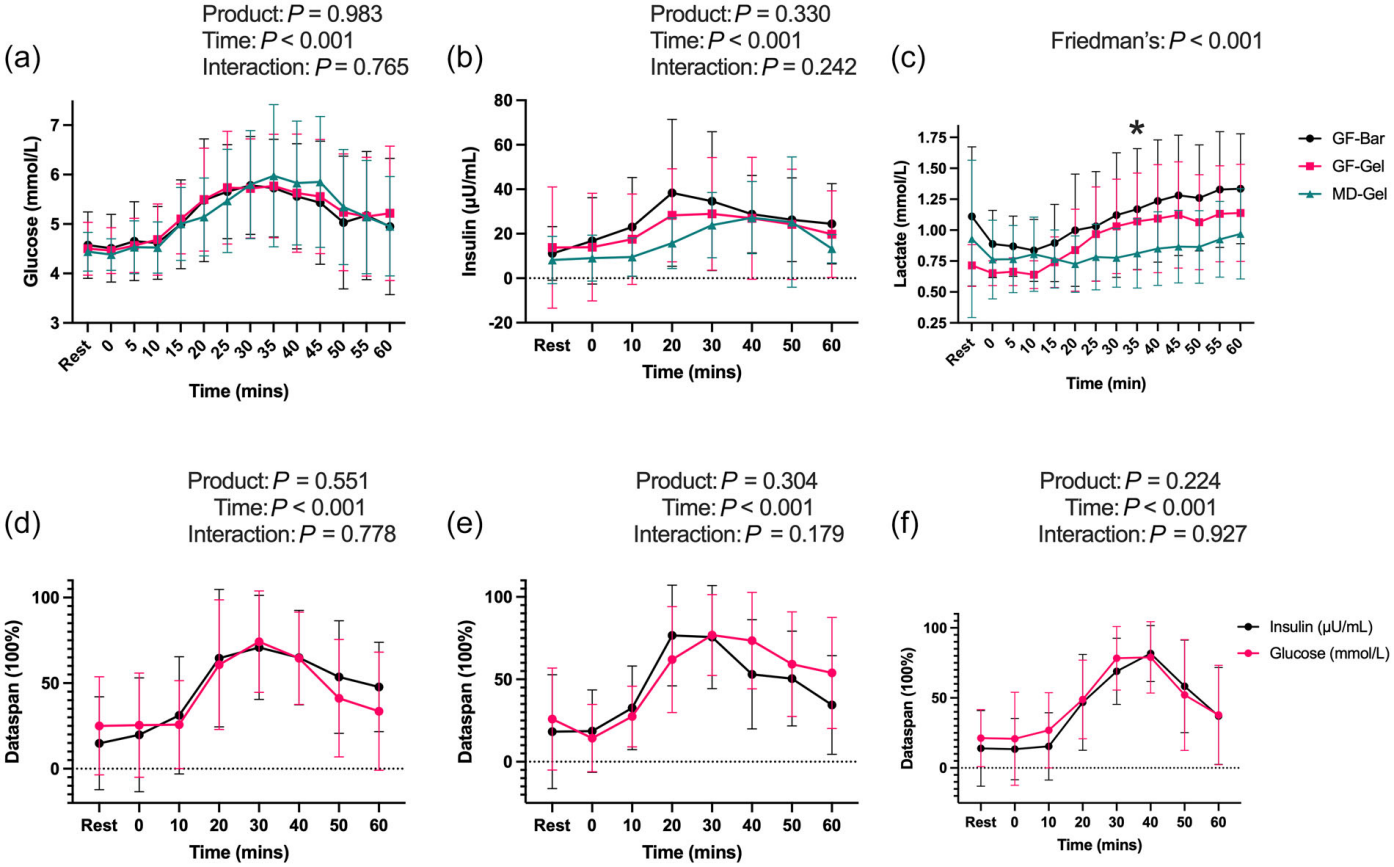
Blood glucose (a) and insulin (b) concentrations were similar. (c) Lactate was significantly greater in the GF‐Bar than MD‐Gel at 35 min. (d–f) Normalised (data span = 100%) mean ± SD comparisons between insulin and glucose in response to 45 g carbohydrate showed no differences between insulin and glucose for GF‐Bar (d), GF‐Gel (e), and MD‐Gel (f) (*P* > 0.05).

A significant main effect of time was found for both glucose (*F*(2.865, 128.9) = 24.62, *P* < 0.001, ηp^2^ = 0.353) and insulin (*F*(3.609, 162.4) = 13.92, *P* < 0.001, ηp^2^ = 0.236). However, there were no significant main effects of product for glucose (*F*(2, 45) = 0.016, *P* = 0.983, ηp^2^ = 0.001) or insulin (*F*(2, 45) = 1.134, *P* = 0.330, ηp^2^  = 0.086). There were no significant interactions between time and product for glucose (*F*(5.73, 128.9) = 0.545, *P* = 0.765, ηp^2^  = 0.023) or insulin (*F*(7.218, 162.4) = 1.32, *P* =  0.242), ηp^2^ = 0.055 (Figure 2). The area under the curve for glucose was similar between products (total area ± standard error) (GF‐Bar 314.3 ± 12.45, GF‐Gel 317.2 ± 12.10, MD‐Gel 316.50 ± 12.04). No significant main effects were seen for mean peak glucose concentration (GF‐Bar 6.59 ± 1.18, GF‐Gel 6.20 ± 1.14 mmol L^−1^, MD‐Gel 6.42 ± 1.15 mmol L^−1^, *F*(1.888, 28.32) = 0.592, *P* = 0.550, ηp^2^  = 0.038) and time to glucose peak (GF‐Bar 31.25 ± 13.96 min, GF‐Gel 39.06 ± 12.28 min, MD‐Gel 33.13 ± 11.09 min, *F*(1.614, 24.21) = 1.856, *P* = 0.182, ηp^2^  = 0.110), reflecting comparable glucose metabolism across the OGTT.

The GF‐Bar elicited a greater area under the curve for insulin (total area ± standard error) (185.8 ± 43.77) than the GF‐Gel (156.7 ± 44.76) and MD‐Gel (121.4 ± 29.35), indicating a greater or more prolonged insulin response when consuming the GF‐Bar.

Friedman's test revealed a significant difference in lactate concentration between the products (χ^2^ (41) = 253, *P* < 0.001). The GF‐Bar had a greater mean rank for lactate concentration (26) than the GF‐Gel (20) and MD‐Gel (17). This was consistent with the raw mean data (GF‐Bar 1.08 ± 0.42 mmol L^−1^, GF‐Gel 0.91 ± 0.30 mmol L^−1^, MD‐Gel 0.81 ± 0.31 mmol L^−1^). Dunn's multiple comparisons revealed a significantly greater lactate concentration in the GF‐Bar than in the MD‐Gel at 35 min (GF‐Bar mean rank = 29, raw data mean = 1.17 ± 0.49 mmol L^−1^, MD‐Gel mean rank = 14, raw data mean = 0.77 ± 0.28 mmol L^−1^, *P* = 0.017, Figure 2c).

Following normalisation, the relative changes in glucose and insulin over time were compared to examine the temporal pattern of glucose and insulin throughout the study period. Both variables were presented as percentage changes from baseline (0%) and plotted for each participant every 10 min across the 1‐h OGTT. This normalisation approach enabled direct comparison of glucose and insulin dynamics independent of individual baseline values. A two‐way repeated measures ANOVA revealed a main effect of time (*P* < 0.001) but no significant product or interaction effect between glucose and insulin concentrations (all *P* > 0.2, Figure 2d).

#### 3.1.2 The glucose–fructose bar enhanced carbohydrate oxidation

**FIGURE 3:**
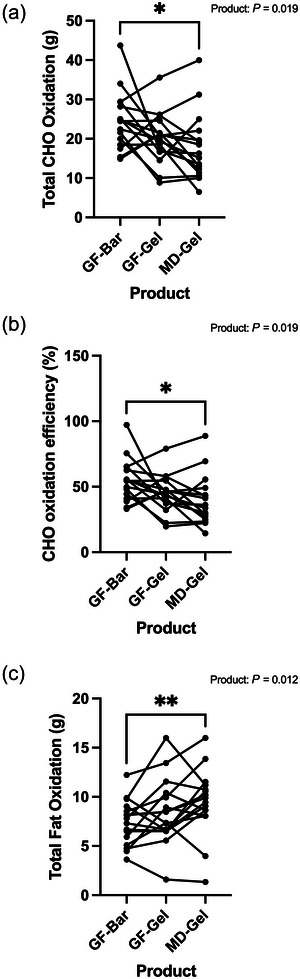
(a) Consuming the GF‐Bar resulted in a significant increase in total carbohydrate oxidation compared to the MD‐Gel in a modified 1‐h OGTT. (b) Carbohydrate oxidation efficiency was significantly greater in the GF‐Bar than the MD‐Gel. (c) Total fat oxidation was suppressed to a greater extent for the GF‐Bar than the MD‐Gel during the 1‐h modified OGTT. *P < 0.05; **P < 0.01.

A one‐way repeated measures ANOVA revealed that total carbohydrate oxidation significantly differed between products (*F*(1.615, 24.23) = 5.09, *P* = 0.019, ηp^2^ = 0.253). Tukey multiple comparisons revealed that the GF‐Bar had significantly greater total carbohydrate oxidation across the 1‐h OGTT than the MD‐Gel (GF‐Bar: 24.63 ± 7.38 g, MD‐Gel: 17.77 ± 8.61 g, *P* = 0.038, Figure 3a), despite matched carbohydrate provision. No differences were observed between the GF‐Gel and GF‐Bar, or GF‐Gel and MD‐Gel (GF‐Gel 20.11 ± 6.41 g, all *P* > 0.1).

A one‐way repeated measures ANOVA revealed carbohydrate oxidation efficiency significantly differed between products (*F*(1.615, 24.23) = 5.09, *P* = 0.019, ηp^2^ = 0.253). Tukey multiple comparisons test showed that the GF‐Bar had a significantly greater carbohydrate oxidation efficiency than the MD‐Gel (GF‐Bar 54.73 ± 16.4 %, MD‐Gel 39.5 ± 19.15%, *P* = 0.038, Figure 3b). No differences were seen between the GF‐Gel and GF‐Bar or the GF‐Gel and MD‐Gel (GF‐Gel 44.68 ± 14.25%, all *P* > 0.1).

**FIGURE 4:**
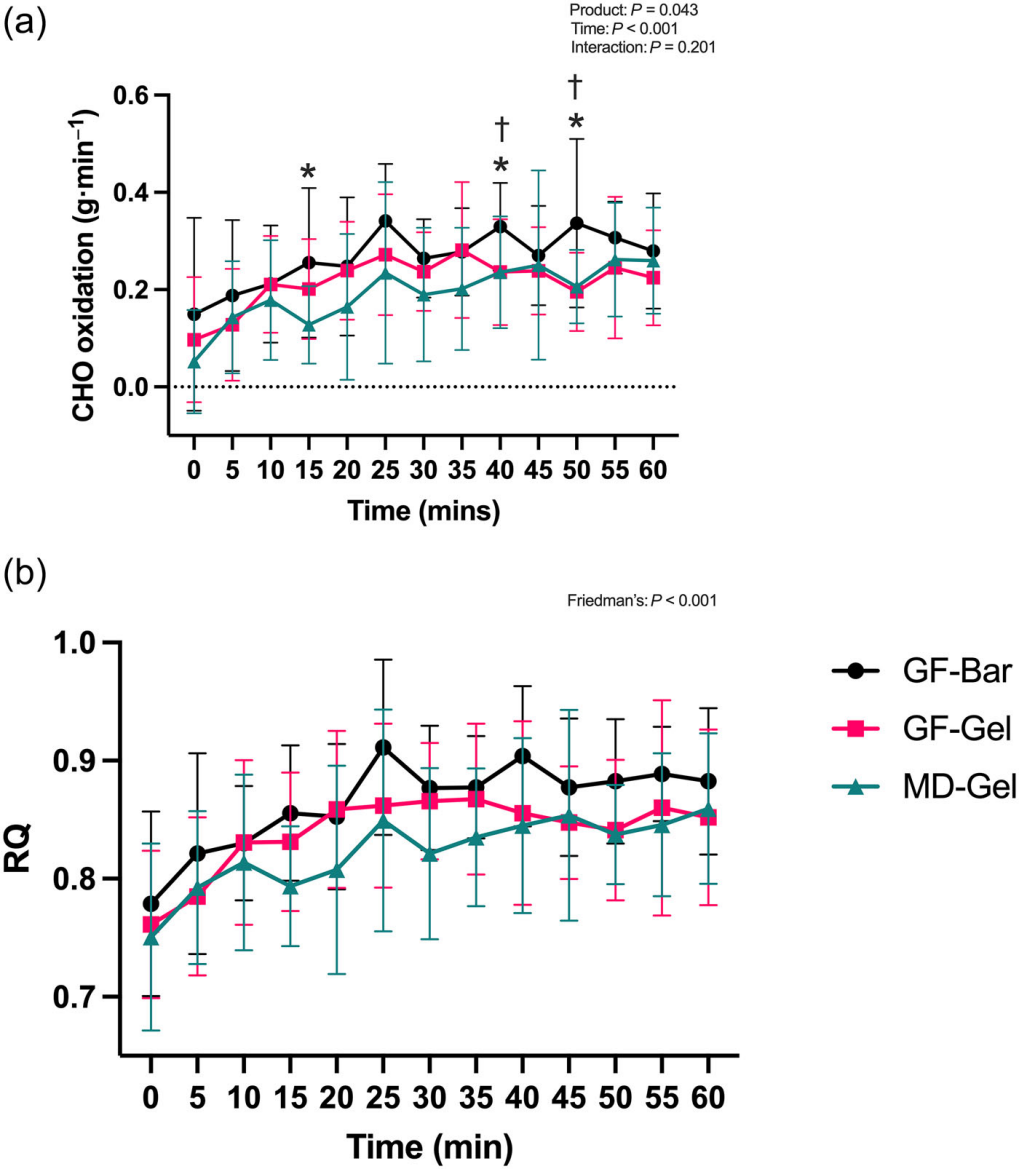
(a) The GF‐Bar elicited significantly greater CHO (carbohydrate) oxidation than the MD‐Gel at 15 min and greater than GF‐Gel and MD‐Gel at both 40 min and 50 min. (b) A higher RQ for the GF‐Bar than the GF‐Gel and MD‐Gel, indicating increased carbohydrate use. *P < 0.05 for GF‐Bar vs. MD‐Gel. †*P* < 0.05 for GF‐Bar vs. GF‐Gel.

A two‐way repeated measures ANOVA revealed a significant main effect for both time (*F*(6.68, 308.7) = 13.98, *P* < 0.001, ηp^2^ = 0.236) and product (*F*(2, 45) = 3.359, *P* = 0.043, ηp^2^ = 0.103) on carbohydrate oxidation per minute, but no interaction effect was present (*F*(13.72, 308.7) = 1.31, *P* = 0.201, ηp^2^ = 0.055), Figure 4a. The GF‐Bar elicited a greater mean carbohydrate oxidation rate (0.27 ± 0.05 g min^−1^) than the GF‐Gel (0.21 ± 0.05 g min^−1^) and MD‐Gel (0.19 ± 0.06 g min^−1^). Tukey multiple comparisons showed that at 15 min, the GF‐Bar had a significantly greater carbohydrate oxidation rate per minute than the MD‐Gel (GF‐Bar 0.25 ± 0.15 g min^−1^; MD‐Gel 0.12 ± 0.07 g min^−1^, *P* = 0.019). At 40 min, the GF‐Bar had a significantly greater carbohydrate oxidation rate per minute than both the GF‐Gel (GF‐Bar 0.32 ± 0.09 g min^−1^; GF‐Gel 0.23 ± 0.10 g min^−1^, *P* = 0.034) and the MD‐Gel (GF‐Bar 0.32 ± 0.09 g min^−1^; MD‐Gel 0.23 ± 0.11 g min^−1^, *P* = 0.041). Similarly, at 50 min, the GF‐Bar's carbohydrate oxidation rate per minute was significantly greater than the GF‐Gel (GF‐Bar 0.33 ± 0.17 g min^−1^; GF‐Gel 0.19 ± 0.08 g min^−1^, *P* = 0.019) and the MD‐Gel (GF‐Bar 0.33 ± 0.17 g min^−1^; MD‐Gel 0.20 ± 0.07 g min^−1^, *P* = 0.030).

#### 3.1.3 Fat oxidation was suppressed to a greater extent in the glucose–fructose bar than the maltodextrin‐gel

In keeping with the carbohydrate oxidation data, a one‐way repeated measures ANOVA showed total fat oxidation significantly differed between products (*F*(1.903, 28.55) = 5.302, *P* = 0.012, ηp^2^ = 0.261). Indeed, total fat oxidation was suppressed to a greater extent in the GF‐Bar than the MD‐Gel (MD‐Gel 9.45 ± 3.41 g, GF‐Bar 7.37 ± 2.29 g, *P* = 0.006, Figure 3c). No differences were observed between GF‐Gel and GF‐Bar or GF‐Gel and MD‐Gel (GF‐Gel 8.46 ± 3.41 g, all *P* > 0.2).

Friedman's test was conducted on the non‐parametric RQ data, revealing significant differences among products (χ^2^ (38) = 187.8, *P* < 0.001, as shown in Figure 4b). Dunn's multiple comparisons revealed no significant differences between products. Mean ranks were as follows: GF‐Bar (24), GF‐Gel (18) and MD‐Gel (16). Raw data means: GF‐Bar (0.86 ± 0.06), GF‐Gel (0.84 ± 0.07) and MD‐Gel (0.83 ± 0.07).

#### 3.1.4 No differences between electrolytes

Potassium (*F*(3.340, 111.6) = 6.005, *P* = 0.0005, ηp^2^ = 0.152) and sodium concentrations (*F*(3.214, 107.4) = 10.93, *P* < 0.001, ηp^2^ = 0.246) changed significantly over time, whereas chloride did not (*F*(1.869, 63.28) = 1.614, *P* = 0.208, ηp^2^ = 0.046). There were no significant main effects of product (all *P* > 0.2) or time × product interactions (all *P* > 0.4).

### 3.2 Exercise study

#### 3.2.1 Performance metrics were comparable between products

**FIGURE 5:**
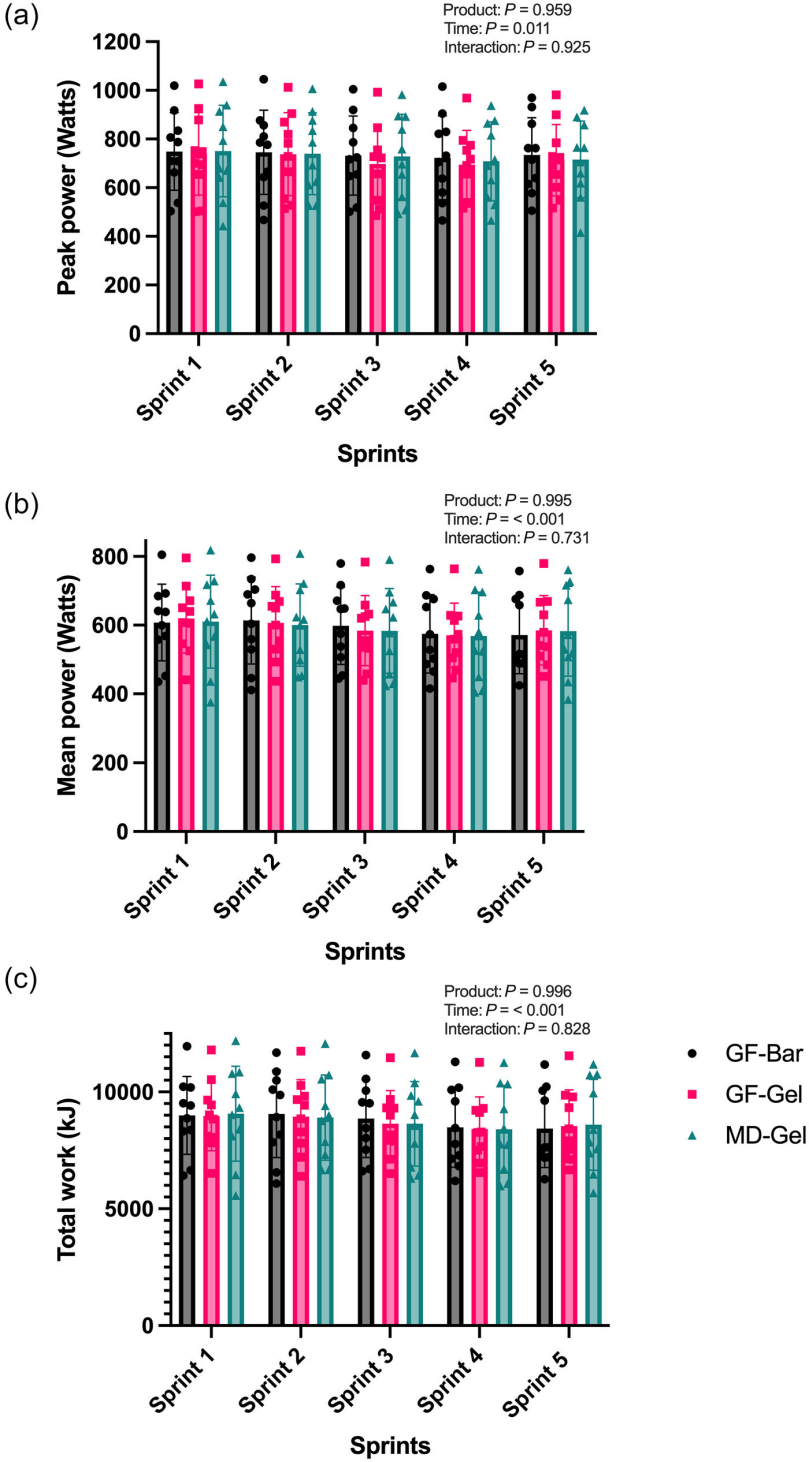
Comparable results in peak power (a), mean power (b), and total work per sprint (c) between the GF‐Bar, GF‐Gel and MD‐Gel.

A two‐way repeated measures ANOVA for peak power per sprint revealed a significant effect of time (*F*(2.814, 75.97) = 4.035, *P* = 0.011, ηp^2^ = 0.130) but no product effect (GF‐Bar 736.7 ± 10.51 W; GF‐Gel 716.4 ± 21.2 W; MD‐Gel 728.5 ± 17.08 W, *F*(2, 27) = 0.040, *P* = 0.959, ηp^2^ = 0.049) or time × product interaction was found (*F*(5.627, 75.97) = 0.303, *P* = 0.925, ηp^2^ = 0.021, Figure 5a). A similar trend was observed for mean power per sprint, where a significant effect for time was found (*F*(2.028, 54.77) = 9.984, *P* ≤ 0.001, ηp^2^ = 0.035), but no difference was detected between products (GF‐Bar 593.1 ± 19.17 W; GF‐Gel 593.1 ± 19.63 W; MD‐Gel 589 ± 16.41 W, *F*(2, 27) = 0.004, *P* = 0.995, ηp^2^ = 0.005) or for the interaction effect (*F*(4.057, 54.77) = 0.508, *P* = 0.731, ηp^2^ = 0.003, Figure 5b). Total work per sprint was also influenced by time (*F*(2.139, 57.74) = 11.23, *P* < 0.001, ηp^2^ = 0.293) but not product (GF‐Bar 8766 ± 290 kJ; GF‐Gel 8701 ± 251 kJ; MD‐Gel 8720 ± 263 kJ, *F*(2,27) = 0.004, *P* = 0.996, ηp^2^ = 0.005) or time × product interaction (*F*(4.057, 54.77) = 0.387, *P* = 0.828, ηp^2^ = 0.027), as depicted in Figure 5c. No significant effect was found for the fatigue index (GF‐Bar 38.6 ± 8.1%; GF‐Gel 38.3 ± 10.0%; MD‐Gel 36.3 ± 9.6%, *F*(1.864, 16.77) = 1.651, *P* = 0.222, ηp^2^ = 0.154).

#### 3.2.2 Similar substrate utilisation and electrolyte responses across carbohydrate products

**FIGURE A1:**
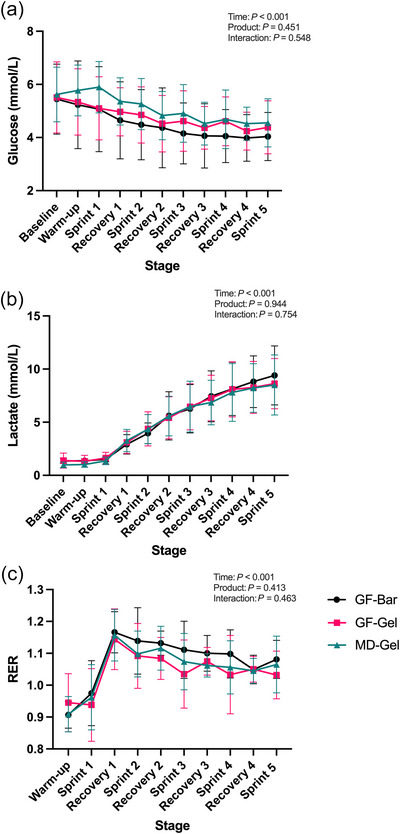
Blood glucose (a), lactate (b), and RER (c) responses during repeated sprints following ingestion of carbohydrate supplements. No significant differences were observed between GF‐Bar, GF‐Gel or MD‐Gel for any variable (*P* > 0.05).

A significant main effect of time was shown for glucose (*F*(3.143, 84.85) = 33.10, *P* < 0.001, ηp^2^ = 0.550), but no effect of product (GF‐Bar 4.5 ± 0.53; GF‐Gel 4.77 ± 0.42; MD‐Gel 5.08 ± 0.52, *F*(2,27) = 0.819, *P* = 0.451, ηp^2^ = 0.249) or time × product interaction was present (*F*(6.285, 84.85) = 0.838, *P* = 0.548, ηp^2^ = 0.058) (Appendix, Figure A1). Similarly, a significant main effect of time was shown for lactate (*F*(1.458, 39.37) = 185.5, *P* < 0.001, ηp^2^ = 0.872), but no effect of product (GF‐Bar 5.16 ± 3.09; GF‐Gel 5.09 ± 2.88; MD‐Gel 4.95 ± 2.92, *F*(2, 27) = 0.912, *P* = 0.944, ηp^2^ = 0.006) or time × product interaction was present (*F*(2.916, 39.37) = 0.391, *P* = 0.754, ηp^2^ = 0.028) (Appendix, Figure A1). RER had a significant main effect of time (*F*(2.187, 59.04) = 42.37, *P* < 0.001, ηp^2^ = 0.610), but not product (GF‐Bar 1.08 ± 0.08; GF‐Gel 1.04 ± 0.06; MD‐Gel 1.05 ± 0.07, *F*(2, 27) = 0.912, *P* = 0.413, ηp^2^ = 0.061) or time × product interaction (*F*(4.373, 59.04) = 0.922, *P* = 0.463, ηp^2^ = 0.063, Figure A1), further supporting comparable substrate utilisation during repeated sprints. A main effect of time was seen for potassium (*F*(3.088, 55.58) = 9.029, *P* < 0.001, ηp^2^ = 0.334), chloride (*F*(3.211, 57.80) = 3.091, *P* = 0.031, ηp^2^ = 0.146), sodium (*F*(3.226, 58.06) = 20.82, *P* < 0.001, ηp^2^ = 0.536) and calcium (*F*(3.574, 64.34) = 15.51, *P* < 0.001, ηp^2^ = 0.462), but no product or time × product interactions were found (all *P* > 0.2, Appendix, Table A1).

#### 3.2.3 Minimal gastrointestinal discomfort and comparable perceptual responses across carbohydrate products

A one‐way repeated measures ANOVA for change in GI discomfort found no significant differences (*F*(1.166, 10.50) = 1.345, *P* = 0.280, ηp^2^ = 0.130), with subjects reporting minimal GI discomfort throughout, demonstrating comparable tolerability with both the volume and compositions of each carbohydrate product (Table A1). Similarly, no significant differences were seen for changes in RPE score (*F*(1.384, 12.46) = 2.684, *P* = 0.119, ηp^2^ = 0.229, Table A1). Heart rate changed over time (*F*(3.574, 64.34) = 15.51, *P* < 0.001, ηp^2^ = 0.462), but there was no effect of product (*F*(2, 27) = 0.485, *P* = 0.620, ηp^2^ = 0.101) or time × product interaction (*F*(7.53, 101.7) = 0.633, *P* = 0.738, ηp^2^ = 0.044, Table A1).

## 4 DISCUSSION

This research found that the GF‐Bar resulted in a higher carbohydrate oxidation rate per minute in a resting state compared to the GF‐Gel and the MD‐Gel. Additionally, the GF‐Bar elicited a significantly greater total carbohydrate oxidation rate and oxidation efficiency than the MD‐Gel. This elevated carbohydrate oxidation at rest may translate to improved exercise performance by enhancing the availability of readily oxidisable fuel during physical activity (Hargreaves & Spriet, 2020). This increased carbohydrate oxidation is likely what led to the significantly greater lactate concentration in the GF‐Bar compared to MD‐Gel, as the greater breakdown of carbohydrates through aerobic glycolysis may have led to more pyruvate being fully oxidised, resulting in excess pyruvate being converted to lactate (Hargreaves & Spriet, 2020). Furthermore, it is known that fructose ingestion in multiple‐transportable carbohydrate solutions can elevate lactate concentration (Jentjens & Jeukendrup, 2005; Jentjens et al., 2004). This is suggested to be due to fructose being rapidly phosphorylated in the liver to fructose‐1‐phosphate by fructokinase, an enzyme with higher activity than hexokinase or glucokinase, which act on glucose. This rapid phosphorylation increases fructose‐1‐phosphate, which can activate pyruvate kinase and promote greater pyruvate production, subsequently elevating lactate production (Jentjens & Jeukendrup, 2005; Jentjens et al., 2004). However, the significant differences in substrate oxidation between products seen in the OGTT did not translate into significant differences in exercise performance nor changes in glycaemia during repeated sprint intervals.

Differences in the carbohydrate formulations and compositions likely influenced these findings. The glucose–fructose compositions in the GF‐Bar and GF‐Gel likely increased carbohydrate oxidation due to the immediate availability of free glucose combined with fructose, utilising both SGLT1 and GLUT5 transporters (Jentjens et al., 2004; Pfeiffer et al., 2010), allowing for rapid oxidation and energy availability. However, it should be noted that the ergogenic benefits of multiple transportable carbohydrate sources are most apparent when consumed in larger volumes (≥90 g h^−1^) during prolonged endurance events (≥2 h) (Jeukendrup, 2010). In contrast, the MD‐Gel relies on maltodextrin, which must be broken down to glucose before absorption (Hofman et al., 2016). Although maltodextrin's absorption rate is not significantly slower than that of glucose (Gonzalez et al., 2017), the MD‐Gel does not benefit from the simultaneous utilisation of multiple transporters, as occurs with the GF‐Bar and GF‐Gel. The GF‐Gel utilises a hydrogel formula comprising three‐dimensional hydrophilic polymers containing sodium alginate and pectin, which aid the absorption of multiple transportable carbohydrates by delivering them gradually at a pH level that is biocompatible with the stomach and intestine (King et al., 2020; Rowe et al., 2022). This may slow the release of sugars into the bloodstream and result in reduced carbohydrate oxidation compared with the GF‐Bar. These differences highlight how formulation and carbohydrate type influence oxidation rate and energy availability.

Furthermore, the greater suppression of fat oxidation rates in the GF‐Bar compared to the MD‐Gel is indicative of increased carbohydrate availability or utilisation. One of the primary functions of carbohydrate supplementation during exercise is to enhance exogenous carbohydrate oxidation and increase glycolytic flux, providing rapid energy to support exercise performance (Baker et al., 2015; Gromova et al., 2021; Podlogar & Wallis, 2022; Podlogar et al., 2022; Rollo et al., 2020). In this context, elevated rates of fat oxidation, as observed with the MD‐Gel, suggest a diminished reliance on carbohydrate as a fuel source, consistent with the lower rates of carbohydrate oxidation shown in the MD‐Gel. This reciprocal shift in substrate use is consistent with the Randle cycle, which describes how increased carbohydrate oxidation suppresses fat oxidation and vice versa, through substrate competition and enzymatic regulation (Spriet, 2014). Given that fat oxidation yields ATP at a slower rate than carbohydrate metabolism, a shift toward greater fat oxidation may impair the delivery of rapid energy and could hinder exercise performance. While indirect calorimetry provides valid estimates of whole‐body carbohydrate and fat oxidation, it does not directly quantify lipolysis or enzymatic flux; therefore, the findings reflect relative substrate shifts rather than direct mechanistic measures. Nonetheless, such reciprocal changes in substrate utilisation are consistent with established models of fuel competition, including the Randle cycle (Frayn, 1983; Jeukendrup & Wallis, 2005; Spriet, 2014).

Although there were metabolic differences during the OGTT, including increased carbohydrate oxidation with the GF‐Bar, these did not translate to improved performance during repeated sprint cycling. This discrepancy likely reflects the energy demands of short‐duration, high‐intensity efforts, predominantly fuelled by phosphocreatine and intramuscular glycogen, rather than circulating glucose or exogenous carbohydrate availability (Vigh‐Larsen et al., 2021). Indeed, previous research demonstrates that carbohydrate supplementation has minimal effect on glycogen depletion or sprint performance during high‐intensity intermittent exercise, even when blood glucose is elevated (Vigh‐Larsen et al., 2024). Therefore, while the GF‐Bar affected substrate utilisation at rest, this did not translate to a functional advantage under the specific demands of our sprint protocol.

During the recovery periods between sprints, fat oxidation likely contributed more to energy provision; however, carbohydrate supplementation may have helped to maintain glycaemia, in addition to increased hepatic glucose output (Hargreaves & Spriet, 2020), potentially delaying the onset of fatigue associated with hypoglycaemia (Cao et al., 2025; Prins et al., 2025). In this aspect, the present study showed similar reductions in performance over the five sprints with all three supplements and no differences in RPE, suggesting that no one product was superior in maintaining performance or reducing the perception of fatigue. Thus, the exogenous effects of carbohydrate supplementation may be more relevant to prolonged or glycogen‐depleting exercise, where maintaining glycaemia or delaying glycogen depletion plays a more critical role (Kuipers et al., 1987; Podlogar et al., 2023; Wallis et al., 2008). Although electrolyte content differed between supplements, the amounts were small and did not meaningfully affect circulating electrolytes, the OGTT, or repeated sprint performance. No subjects reported significant GI discomfort during repeated sprints after consuming any of the three carbohydrate supplements, each providing 45 g of carbohydrate.

### 4.1 Limitations

Full blinding of subjects was not possible due to the differing physical forms of the supplements, making them distinguishable upon consumption. This could have influenced subjective outcomes like GI discomfort and RPE, as subjects may be differentially affected by the composition (solid, semi‐solid, liquid) (Guillochon & Rowlands, 2017). However, research demonstrates little difference in GI discomfort and RPE between solid, semi‐solid or liquid carbohydrate sources (Hearris et al., 2022). While the carbohydrate content of each supplement was matched, the absolute quantities of glucose and fructose for GF‐Bar and GF‐Gel were not publicly available. Further, we did not control for the differences in volume of water consumed with carbohydrate (i.e., small water content differences between GF‐Bar, GF‐Gel and MD‐Gel), which may have impacted gastrointestinal kinetics and substrate availability. Subjects were unaware of product names or formulations before consumption, and the researcher remained blinded until all analysis was completed. Furthermore, the absence of a control may limit interpretation, but the research aim was to directly compare the physiological responses between commercially available carbohydrate supplements. The study also used antegrade rather than retrograde venous cannulation. Antegrade cannulation is a less invasive method with minimal impact on metabolite measurement accuracy (Wrench et al., 2024).

A limitation of this study is that substrate oxidation was estimated from indirect calorimetry (Frayn, 1983), with RQ reflecting the balance of carbohydrate and fat oxidation. While an upward shift in RQ indicates greater reliance on carbohydrate at the expense of fat, consistent with the Randle cycle (Spriet, 2014), indirect calorimetry cannot distinguish processes such as glycogenolysis or lipolysis. Future work may look to use stable isotope tracers, such as ^13^C‐labelled carbohydrates, which may offer more accurate insights into exogenous carbohydrate and fat oxidation (Davies, 2020). Further, without tracers, it is not possible to distinguish between exogenous and endogenous oxidation, potentially reducing the accuracy of metabolic assessments (Gonzalez & King, 2022). While cost constraints prevented their use, future research should incorporate stable isotopes to better assess substrate utilisation following the GF‐Bar, GF‐Gel and MD‐Gel ingestion. A further limitation is the lack of direct measurement of muscle glycogen, which would have provided a clearer understanding of exogenous glycogen utilisation and recovery in response to carbohydrate supplementation. Future studies incorporating muscle biopsies could enhance the confidence and precision of interpretations relating to substrate utilisation. Only males were recruited for the OGTT to minimise the variability from sex differences in substrate oxidation and insulin responses (Cano et al., 2022; Sanchez et al., 2024). Our approach focused on the detection of subtle metabolic differences between the carbohydrate supplements. While this may limit generalisability to female athletes, the exercise study included both sexes to reflect real‐world sporting contexts better. The repeated sprint protocol, while appropriate for assessing performance outcomes, offers limited insight into substrate oxidation due to its reliance on anaerobic energy systems. Additionally, the small number of participants in each sex subgroup limits the ability to draw robust conclusions regarding sex‐based differences.

### 4.2 Conclusion

The findings of this study show that the GF‐Bar elicits greater carbohydrate oxidation at rest compared to the GF‐Gel and MD‐Gel, providing athletes with an effective alternative for carbohydrate supplementation. However, these metabolic differences did not enhance repeated sprint cycling performance. These results suggest that the ergogenic effects of acute carbohydrate supplementation are exercise‐specific, with limited influence on short‐duration, intermittent efforts, and contribute to clarifying the previously mixed findings on carbohydrate availability and high‐intensity exercise.

## AUTHOR CONTRIBUTIONS

Ewan Dean contributed to conceptualisation, methodology, investigation, data curation, formal analysis, visualisation, project administration, writing – original draft, and writing – review and editing. Ash Osborne contributed to project administration and writing – review and editing. Daren Subar contributed to resources, supervision, and writing ‐ review and editing. Paul Hendrickse contributed to conceptualisation, supervision, and writing – review and editing. Christopher J. Gaffney contributed to conceptualisation, funding acquisition, resources, supervision, and writing – review and editing. All authors have read and approved the final version of this manuscript and agree to be accountable for all aspects of the work in ensuring that questions related to the accuracy or integrity of any part of the work are appropriately investigated and resolved. All persons designated as authors qualify for authorship, and all those who qualify for authorship are listed.

## CONFLICT OF INTEREST

The author discloses receipt of funding from Omega EFA Ltd (trading as Team Nutrition), a company with potential commercial interests in the subject matter of this research. This relationship has been fully disclosed, and appropriate measures are in place to manage any potential conflicts of interest.

## Data Availability

Data generated or analysed during this study are provided in full within the published article.

## 1

**TABLE A1:** Physiological and metabolic responses under the GF‐Bar, GF‐Gel and MD‐Gel during repeated sprints.

	GF‐Bar	GF‐Gel	MD‐Gel	*P*
Potassium (mmol L^−1^)	4.66 ± 0.23	4.87 ± 0.34	4.64 ± 0.30	0.567
Chloride (mmol L^−1^)	106.8 ± 0.75	106.3 ± 0.47	106.3 ± 0.64	0.758
Sodium (mmol L^−1^)	13.9 ± 2.10	139.7 ± 1.08	140.3 ± 1.71	0.758
Calcium (mmol L^−1^)	1.18 ± 0.03	1.21 ± 0.01	1.19 ± 0.02	0.367
Heart rate (bpm)	126 ± 21	129 ± 20	131 ± 21	0.620
ΔGI	0.7 ± 1.06	0.6 ± 1.08	0.3 ± 0.67	0.280
ΔRPE	9.3 ± 1.5	8.2 ± 2.6	9.5 ± 1.4	0.119

ΔGI, change in gastrointestinal discomfort by a modified version of the Gastrointestinal Symptom Rating Scale; ΔRPE, change in rating of perceived exertion.
